# Genetic diversity of Imjin virus in the Ussuri white-toothed shrew (*Crocidura lasiura*) in the Republic of Korea, 2004-2010

**DOI:** 10.1186/1743-422X-8-56

**Published:** 2011-02-08

**Authors:** Se Hun Gu, Hae Ji Kang, Luck Ju Baek, Ji Yun Noh, Heung-Chul Kim, Terry A Klein, Richard Yanagihara, Jin-Won Song

**Affiliations:** 1 Department of Microbiology, College of Medicine, and Institute for Viral Diseases, Korea University, 5-ga, Anam-dong, Sungbuk-gu, Seoul 136-705, Korea; 2 Departments of Pediatrics and Tropical Medicine, Medical Microbiology and Pharmacology, John A. Burns School of Medicine, University of Hawai'i at Manoa, 651 Ilalo Street, Honolulu, HI 96813, USA; 3 Force Health Protection and Preventive Medicine, 65th Medical Brigade, U.S. Army MEDDAC-Korea, Unit 15281, APO AP 96205-5281, USA

## Abstract

Recently, Imjin virus (MJNV), a genetically distinct hantavirus, was isolated from lung tissues of the Ussuri white-toothed shrew (*Crocidura lasiura*) captured near the demilitarized zone in the Republic of Korea. To clarify the genetic diversity of MJNV, partial M- and L-segment sequences were amplified from lung tissues of 12 of 37 (32.4%) anti-MJNV IgG antibody-positive Ussuri white-toothed shrews captured between 2004 and 2010. A 531-nucleotide region of the M segment (coordinates 2,255 to 2,785) revealed that the 12 MJNV strains differed by 0-12.2% and 0-2.3% at the nucleotide and amino acid levels, respectively. A similar degree of nucleotide (0.2-11.9%) and amino acid (0-3.8%) difference was found in a 632-nucleotide length of the L segment (coordinates 962 to 1,593) of nine MJNV strains. Phylogenetic analyses, based on the partial M and L segments of MJNV strains generated by the neighbor-joining and maximum likelihood methods, showed geographic-specific clustering, akin to the phylogeography of rodent-borne hantaviruses.

## Findings

Genetically distinct hantaviruses have been discovered recently in several species of shrews (Order Soricomorpha, Family Soricidae) across four continents, including the Eurasian common shrew (*Sorex araneus*) [1], Chinese mole shrew (*Anourosorex squamipes*) [2], Therese's shrew (*Crocidura theresae*) [3], masked shrew (*Sorex cinereus*) [4], dusky shrew (*Sorex monticolus*) [4], northern short-tailed shrew (*Blarina brevicauda*) [5] and flat-skulled shrew (*Sorex roboratus*) [6]. Also, a novel hantavirus, named Imjin virus (MJNV), has been isolated from tissues of the Ussuri white-toothed shrew (*Crocidura lasiura*) captured in the Republic of Korea [7]. As demonstrated recently, Seewis virus (SWSV) harbored by the Eurasian common shrew throughout its broad geographic range exhibits geographic-specific genetic variation, similar to the phylogeography of rodent-borne hantaviruses [8,9]. A U.S. Army surveillance program, aimed at monitoring the prevalence of Hantaan virus (HTNV) infection in striped field mouse (*Apodemus agrarius*) populations near the demilitarized zone (DMZ) in Korea, provided an opportunity to investigate the genetic diversity and phylogeography of MJNV in the crocidurine shrew species reservoir.

**Figure 1:**
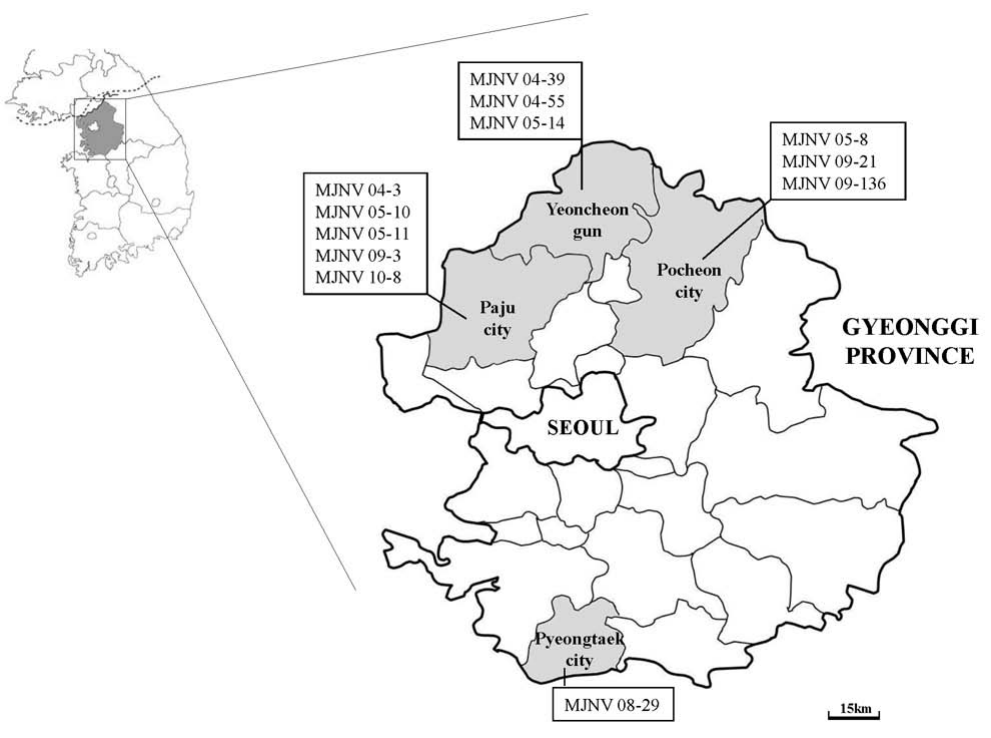
**Map of Paju City, Yeoncheon County, and Pocheon City near the DMZ, showing the locations of the trap sites in Gyeonggi province**. MJNV RT-PCR-positive Ussuri shrews (boxes) were trapped in three cities and/or counties (gun), in the Republic of Korea during 2004-2010.

A total of 466 Ussuri white-toothed shrews were live caught in multiple sites, located 30 to 370 kilometers from Seoul, during March 2004 to May 2010 (Figure 1): Paju City, Yeoncheon-gun, Pocheon City, Dongducheon City, Yangpyeong-gun, Osan City, Suwon City and Pyeongtaek City in Gyeonggi province; Sincheorwon-gun and Pyeongchang-gun in Gangwon province; Gunsan City in Jeollabuk province; and Haenam-gun in Jeollanam province [10-12]. Sera collected from *C. lasiura *were initially diluted 1:32 in phosphate buffered saline and examined for IgG antibodies against MJNV, using the indirect immunofluorescent antibody (IFA) test [7]. In the absence of a crocidurine shrew species-specific secondary antibody, an equal mixture of fluorescein isothiocyanate-conjugated goat anti-mouse and rat IgG (ICN Pharmaceuticals, Inc., Aurora, OH) was employed.

Total RNA was extracted from lung tissues of anti-MJNV IgG antibody-positive shrews, using the RNA-Bee™ isolation kit (TEL-TEST, Inc., Friendswood, TX), and reverse transcribed, using M-MLV reverse transcriptase (Promega, Madison, WI) and a universal primer (OSM55: 5'-TAGTAGTAGACTCC-3'). Polymerase chain reaction (PCR) was performed in 50-μL mixtures, containing 200 μM dNTP, 0.5 U of SuperTherm Taq DNA polymerase (JMR Holdings, London, UK), 1 μg of cDNA and 5 pM of each primer. Oligonucleotide primer sequences for nested PCR were MJN-M2235F: 5'-CATGGAAGAGTGCAACATGT-3' and MJN-M2855R: 5'-TATGGTCCCTAGATGTACT-3', MJV-M2235F and MJN-M2805R: 5'-TCTATAATAGGATCAGTCAT-3' for the M segment; MJN-L942F: 5'-TCAGAATATAAACCTATGAC-3' MJN-L1636R: 5'-GATCAACAAACCCATATC-3', then MJN-L942F and MJN-L1612R: 5'-CTTACATGAGCAGTGGCTA-3' for the L segment. Initial denaturation was at 94°C for 5 min, followed by 15 cycles of denaturation at 94°C for 40 sec, annealing at 50°C for 40 sec, elongation at 72°C for 1 min, then 25 cycles of denaturation at 94°C for 40 sec, annealing at 52°C for 40 sec and elongation at 72°C for 1 min, in a Mastercycler ep gradient S (Eppendorf AG, Hamburg, Germany).

PCR products were purified by the QIAquick PCR Purification Kit (QIAGEN Inc., Chatsworth, CA), and DNA sequencing was performed in both directions of each PCR product, using the Big-Dye^® ^Terminator v3.1 cycle sequencing kit (Applied Biosystems, Foster City, CA) on an automated sequencer (Model 3730, Applied Biosystems, Foster City, CA) [1,13]. For phylogenetic analysis, the neighbor-joining (N-J), maximum likelihood and MrBayes methods were employed [14]. Genetic distances were computed by PAUP (Phylogenetic Analysis Using Parsimony, version 4.0b), and topologies were evaluated by bootstrap analysis of 1,000 iterations [14].

Host identification of shrews infected with MJNV was confirmed by amplification and sequencing of the cytochrome *b *gene of mitochondrial DNA (mtDNA) using previously described universal primers [7,15].

Sera from 37 of 466 (7.9%) Ussuri white-toothed shrews contained IgG antibodies against MJNV by the IFA test (Table 1). Of 242 male and 224 female Ussuri shrews, anti-MJNV IgG antibodies were found in sera of 20 males (8.3%) and 17 females (7.5%). Infection rates in shrews from the northern part of Gyeonggi province (Paju City: 10.7%, 6/56; Yeoncheon-gun: 19.4%, 14/72; Pocheon City: 6.9%, 9/130) were higher than rates in the southern part (Yangpyeong-gun: 0%, 0/1; Osan City: 0%, 0/2; Suwon City: 0%, 0/1; and Pyeongtaek City: 4.5%, 8/179). No seropositive shrews were captured in Sincheorwon-gun (0%, 0/7) and Pyeongchang-gun (0%, 0/1) in Gangwon province; Gunsan City (0%, 0/2) in Jeollabuk province; and Haenam-gun (0%, 0/10) in Jeollanam province.

**Table 1:** Prevalence of MJNV infection among *Crocidura lasiura *captured in the Republic of Korea, 2004-2010

Trapping year	No. trapped	No. seropositive (%)	RT-PCR (%)
2004	74	11 (14.9)	3 (27.3)
2005	41	7 (17.1)	4 (57.1)
2006	13	0	0
2007	41	4 (9.8)	0
2008	100	5 (5.0)	1 (20.0)
2009	185	9 (4.9)	3 (33.3)
2010	12	1 (8.3)	1 (100)

Total	466	37 (7.9)	12 (32.4)

Of the 37 anti-MJNV IgG antibody-positive Ussuri shrews, MJNV RNA was detected in 12 (nine males and three females) by RT-PCR. This difference in gender-specific prevalence was not statistically significant (Fisher's exact test P = 0.09), possibly because of the small sample size of infected shrews. However, the higher proportion of MJNV RNA-positive male shrews was reminiscent of the over-representation of hantavirus infection in male Norway rats [16], male deer mice [17,18], and male marsh rice rats [19].

**Figure 2:**
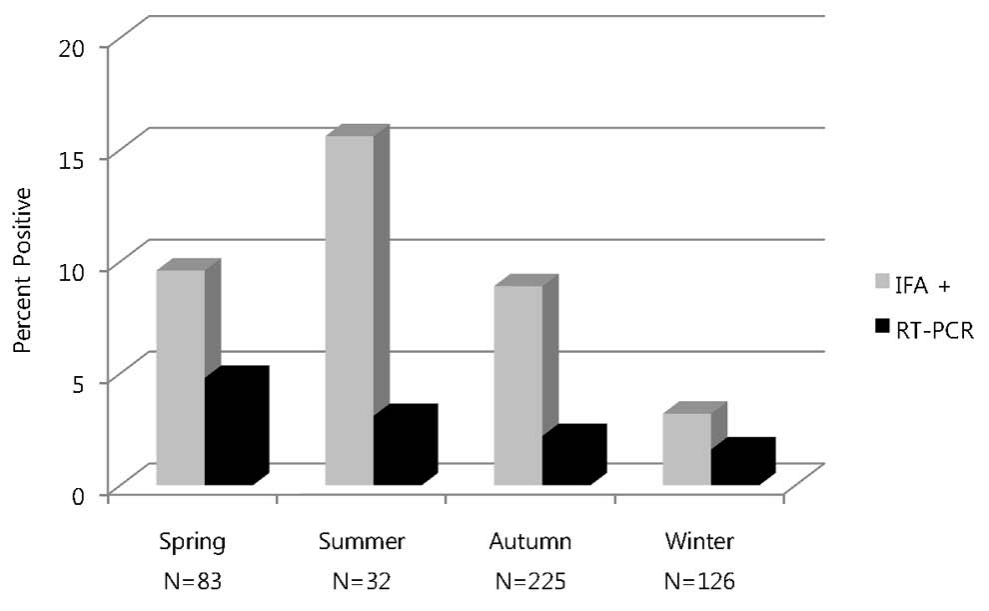
**Comparative seasonal seroprevalence of MJNV infection, as determined by IFA and RT-PCR, in *Crocidura lasiura *captured in the Republic of Korea during the spring, summer, autumn and winter of 2004-2010**.

All 12 MJNV strains were detected in shrews captured in Gyeonggi province: five in Paju City (04-3, 05-10, 05-11, 09-3, 10-8), three in Yeoncheon-gun (04-39, 04-55, 05-14), three in Pocheon City (05-8, 09-21, 09-136), and one in Pyeongtaek City (08-29) (Figure 1, Table 2). Prevalence of MJNV infection, as determined by IFA and RT-PCR, was highest during the summer and spring, respectively (Figure 2), but only a few shrews were captured during the summer compared to other seasons.

**Table 2:** MJNV strains detected in *Crocidura lasiura *in the Republic of Korea

MJNV strain	Trapping site	Trapping date	**GenBank accession no**.	
			
			M segment	L segment
04-3	Dagmar North 2-9/Paju, Gyeonggi	Mar 24, 2004	EF641797†	HQ201409*
04-39	LTA 130-1a/Yeoncheon, Gyeonggi	Dec 4, 2004	EF641802†	HQ201410*
04-55	FP131A-1a/Yeoncheon, Gyeonggi	Dec 7, 2004	EF641799†	EF641807†
05-8	MPRC-7a/Pocheon, Gyeonggi	Sep 10, 2005	EF641800†	HQ201411*
05-10	Dagmar North-7a/Paju, Gyeonggi	Sep 12, 2005	EF641801†	ND
05-11	Story Impact-11a/Paju, Gyeonggi	Sep 14, 2005	EF641798†	EF641806†
05-14	LTA 320/Yeoncheon, Gyeonggi	Nov 9, 2005	EF641803†	HQ201412*
08-29	CP Humphrey/Pyeongtaek, Gyeonggi	Apr 2, 2008	HQ201404*	ND
09-21	Nightmare Range/Pocheon, Gyeonggi	Jun 24, 2009	HQ201405*	ND
09-136	Nightmare Range/Pocheon, Gyeonggi	Oct 28, 2009	HQ201406*	HQ201413*
09-3	Maji-ri/Paju, Gyeonggi	Apr 17, 2009	HQ201407*	HQ201414*
10-8	Dragon Head/Paju, Gyeonggi	May 12, 2010	HQ201408*	HQ201415*

ND, not determined because of insufficient sequence data.

* The GenBank accession numbers (HQ201404-HQ201415) for the MJNV sequences determined in this study.

† The reference sequence (Song et al.) [7]

Overall genetic analysis of a 531-nucleotide region (coordinates 2,255 to 2,785) of the Gc glycoprotein-encoding M segment revealed that MJNV strains from Korea differed by 0-12.2%. However, the amino acid sequences were highly conserved (0-2.3%) (Table 3). This degree of genetic diversity was higher than that of HTNV strains among striped field mice in Korea [20]. In a 632-nucleotide region of the L segment (coordinates 962 to 1,593), MJNV strains differed by 0.2-11.9% and 0-3.8% at the nucleotide and amino acid levels, respectively (Table 4). This level of interstrain difference for the MJNV L segment was similar with that of SWSV strains in Eurasian common shrews from Russia [9]. The genetic distance between MJNV strains and other crocidurine and soricine shrew-borne hantaviruses, including Thottapalayam virus (TPMV) and Cao Bang virus (CBNV), based on the M segment was 26.9 to 42.2% and 16.9 to 50.8% at the nucleotide and amino acid levels, respectively, and the nucleotide and amino acid distance based on the L segment was 28.5 to 50.5% and 31.9 to 61.0%, respectively.

**Table 3:** Percent nucleotide and amino acid sequence similarities of partial M segment of MJNV* compared with other hantaviruses

Strain	MJN 04-3	MJN 04-39	MJNV 04-55	MJN 05-8	MJN 05-10	MJN 05-11	MJN 05-14	MJN 08-29	MJN 09-3	MJN 09-21	MJN 09-136	MJN 10-8	TPM	CBN	HTN	SEO	PUU	SN
MJN 04-3		88.4	98.5	98.7	100	98.5	98.5	98.3	98.5	98.1	98.1	99.4	73.1	58.9	58.4	58.9	60.2	58.9
MJN 04-39	98.3		88.1	88.1	88.3	88.1	88.1	87.9	88.1	87.8	87.8	88.5	72.5	59.1	58.1	57.6	60.6	61.2
MJNV 04-55	100	98.3		98.7	98.5	100	100	99.8	100	98.1	98.1	98.7	72.5	59.3	58.6	59.7	60.6	58.9
MJN 05-8	100	98.3	100		98.7	98.7	98.7	98.5	98.7	98.3	98.3	98.9	72.5	58.9	58.2	59.1	59.6	58.9
MJN 05-10	100	98.3	100	100		98.5	98.5	98.3	98.5	98.1	98.1	99.4	73.1	58.9	58.4	58.9	60.2	58.9
MJN 05-11	100	98.3	100	100	100		100	99.8	100	98.1	98.1	98.7	72.5	59.3	58.6	59.7	60.6	58.9
MJN 05-14	99.4	97.7	100	100	100	100		99.8	100	98.1	98.1	98.7	72.5	59.3	58.6	59.7	60.6	58.9
MJN 08-29	100	98.3	99.4	99.4	99.4	99.4	99.4		99.8	97.9	97.9	98.5	72.3	59.1	58.4	59.5	60.4	58.7
MJN 09-3	100	98.3	100	100	100	100	100	99.4		98.1	98.1	98.7	72.5	59.3	58.6	59.7	60.6	58.9
MJN 09-21	99.4	97.7	99.4	99.4	99.4	99.4	99.4	98.9	99.4		100	98.3	72.3	58.4	57.8	58.6	59.5	58.5
MJN 09-136	99.4	97.7	99.4	99.4	99.4	99.4	99.4	98.9	99.4	100		98.3	72.3	58.4	57.8	58.9	59.5	58.5
MJN 10-8	100	98.3	100	100	100	100	100	99.4	100	99.4	99.4		72.7	58.8	58.2	58.8	60.0	59.2
TPM	82.5	83.1	82.5	82.5	82.5	82.5	82.5	81.9	82.5	81.9	81.9	82.5		57.8	56.9	58.8	60.2	57.0
CBN	49.7	49.2	49.7	49.7	49.7	49.7	49.7	49.2	49.7	49.2	49.2	49.7	52.0		67.4	69.7	66.1	65.3
HTN	53.7	53.1	53.7	53.7	53.7	53.7	53.7	53.1	53.7	53.1	53.1	53.7	53.7	71.8		76.3	68.0	65.0
SEO	52.5	52.0	52.5	52.5	52.5	52.5	52.5	52.0	52.5	52.0	52.0	52.5	53.1	71.8	83.1		67.2	62.3
PUU	59.1	58.5	59.1	59.1	59.1	59.1	59.1	58.5	59.1	58.5	58.5	59.1	55.7	64.8	67.0	67.0		71.8
SN	55.1	54.5	55.1	55.1	55.1	55.1	55.1	55.1	55.1	54.5	54.5	55.1	54.0	67.6	68.2	65.3	69.0	

*MJNV strains 04-3, 05-10, 05-11, 09-3, 10-8 from Paju City; MJNV strains 04-39, 04-55, 05-14 from Yeoncheon-gun; MJNV strains 05-8, 09-21, 09-136 from Pocheon City; MJN strain 08-29 from Pyeongtaek City.

Amino acid similarity shown on bottom triangle and nucleotide similarity shown on top triangle.

**Table 4:** Percent nucleotide and amino acid sequence similarities of partial L segment of MJNV* compared with other hantaviruses

Strain	MJN 04-3	MJN 04-39	MJN 04-55	MJN 05-8	MJN 05-11	MJN 05-14	MJN 09-21	MJN 09-136	MJN 10-8	TPM	SWS	HTN	SEO	PUU	SN
MJN 04-3		89.1	88.8	97.9	98.7	89.4	98.6	98.4	99.2	71.2	49.9	52.9	50.7	54.4	52.4
MJN 04-39	98.6		98.4	88.9	88.4	99.4	88.9	88.8	88.8	70.2	51.4	53.4	51.0	54.8	54.2
MJN 04-55	97.1	97.6		88.6	88.1	99.1	88.6	88.4	88.4	69.4	50.9	52.6	50.7	54.2	52.5
MJN 05-8	100	98.6	97.1		97.6	89.2	98.7	98.9	97.6	71.2	51.0	52.6	51.0	54.4	52.2
MJN 05-11	99.0	97.6	96.2	99.0		88.8	98.3	98.1	98.4	70.8	50.2	52.5	51.2	54.4	51.9
MJN 05-14	99.0	99.5	98.1	99.0	98.1		89.2	89.1	89.1	71.2	50.2	53.4	51.0	55.0	53.0
MJN 09-21	100	98.6	97.1	100	99.0	99.0		99.8	98.3	70.5	50.4	52.8	50.9	54.7	51.9
MJN 09-136	100	98.6	97.1	100	99.0	99.0	100		98.1	70.7	50.4	52.6	50.7	54.5	51.7
MJN 10-8	99.5	98.1	96.7	99.5	98.6	98.6	99.5	99.5		71.5	50.2	53.1	50.7	53.9	52.5
TPM	67.6	67.6	66.7	67.6	67.6	68.1	67.6	67.6	67.1		50.5	51.7	50.9	55.2	52.8
SWS	39.5	40.5	39.0	39.5	39.5	40.0	39.5	39.5	39.5	62.2		63.2	61.5	56.8	58.7
HTN	42.9	42.4	41.9	42.9	42.4	42.9	42.9	42.9	42.9	43.1	55.9		70.2	58.2	57.6
SEO	41.0	41.0	40.0	41.0	40.5	41.0	41.0	41.0	41.0	38.4	58.2	77.9		58.2	59.3
PUU	45.7	45.2	44.8	45.7	45.7	45.7	45.7	45.7	45.2	46.4	53.1	54.0	77.9		64.6
SN	45.7	46.7	45.2	45.7	45.7	46.2	45.7	45.7	45.7	46.4	54.9	54.5	55.4	64.8	

*MJNV strains 04-3, 05-11, 10-8 from Paju City; MJNV strains 04-39, 04-55, 05-14 from Yeoncheon-gun; MJNV strain 05-8, 09-21, 09-136 from Pocheon City.

Amino acid similarity shown on bottom triangle and nucleotide similarity shown on top triangle.

**Figure 3:**
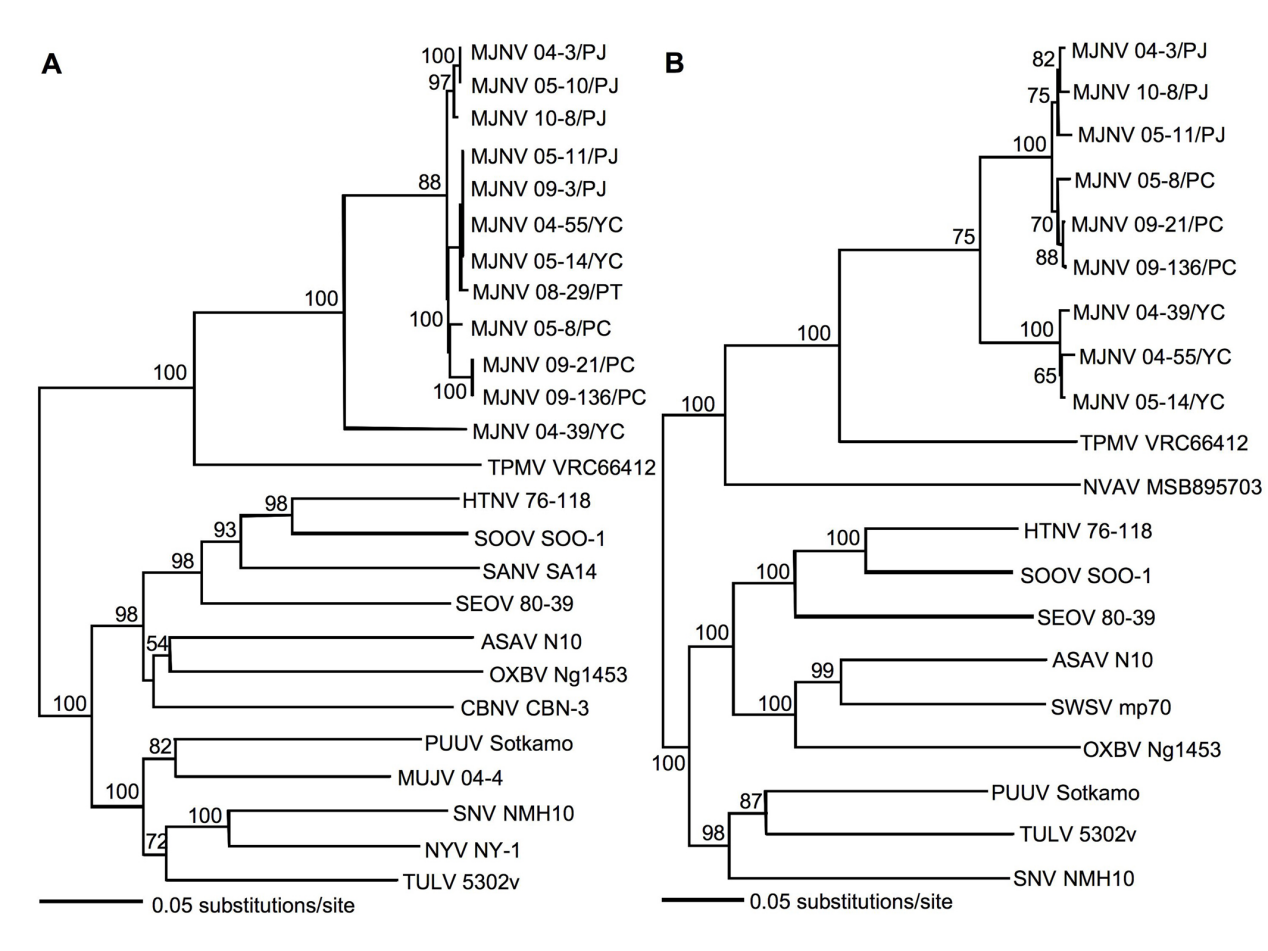
**Phylogenetic trees generated by the neighbor-joining method using PAUP version 4.0b, based on the (A) 531-nucleotide M segment and (B) 632-nucleotide L segment of MJNV strains**. The phylogenetic positions of MJNV strains are shown in relationship to representative soricomorph- and rodent-borne hantaviruses, including Thottapalayam virus (TPMV VRC66412; EU001329, EU001330), Cao Bang virus (CBNV 3; EF543525, EF543526), Asama virus (ASAV N10; EU929075, EU929078), Oxbow virus (OXBV Ng1453; FJ539167, FJ593497), Seewis virus (SWSV mp70; EF636026), Nova virus (NVAV MSB95703; FJ593498), Hantaan virus A(HTNV 76-118; NC_005219, NC_005222), Soochong virus (SOOV SOO-1; AY675353, DQ056292), Sangassou virus (SANV SA14; DQ268651), Seoul virus (SEOV 80-39; NC_005237, NC_005238), Puumala virus (PUUV Sotkamo; NC_005223, NC_005225), Muju virus (MUJV 04-4, EF198413), Tula virus (TULV 5302v; NC_005228, NC_005226), New York virus (NYV NY-1; U36802) and Sin Nombre virus (SNV NMH10; NC_005215, NC_005217).

Phylogenetic analyses of partial L-segment sequences of MJNV strains, using the N-J, maximum likelihood and Bayesian methods, showed geographic-specific genetic variation (Figure 3). In the N-J tree based on the M segment, MJNV strain 04-39 from Yeoncheon-gun, Gyeonggi province, was the most divergent. This was similar to the phylogeography of rodent-borne hantaviruses, including HTNV in *Apodemus agrarius *[20], Soochong virus in *Apodemus peninsulae *[13], Puumala virus in *Myodes glareolus *[21,22], Muju virus in *Myodes regulus *[23] and Tula virus in *Microtus arvalis *[24]. Moreover, as recently shown for SWSV in *Sorex araneus *[8,9], the geographic-specific genetic variation of MJNV suggested a long-standing virus-host relationship between MJNV and *C. lasiura*, with local host-specific adaptation.

Genetic studies of MJNV in Ussuri white-toothed shrews captured beyond Korea, in northern China and far eastern Russia, are warranted to further explore if host sharing exists for MJNV among sympatric and syntopic crocidurine shrews. Also, future research on the genetic diversity of other hantaviruses harbored by shrew species that have broad geographic distributions (such as prototype TPMV carried by the Asian house shrew) might provide additional insights into the phylogeography and evolutionary history of hantaviruses and their soricid hosts.

## Competing interests

The authors declare that they have no competing interests.

## Authors' contributions

SHG performed the RNA extraction, RT-PCR and DNA sequencing. HJK provided the preliminary sequencing data and assisted with the phylogenetic analysis. LJB, HCK and TAK arranged the field expeditions and provided the background data on wild-caught shrews. JYN performed the genetic analysis, RY arranged the collaboration and provided scientific oversight, and JWS conceived the research design, coordinated the implementation of the project, including the design of oligonucleotide primers and phylogenetic analysis. All authors read and approved the final manuscript.
